# Inadvertent Percutaneous Endoscopic Gastrostomy Tube Placement through the Transverse Colon to the Stomach Causing Intractable Diarrhea: A Case Report

**DOI:** 10.1155/2011/849460

**Published:** 2011-12-20

**Authors:** David T. Burke, Andrew I. Geller, Alexios G. Carayannopoulos, Richard Goldstein

**Affiliations:** ^1^Department of Physical Medicine and Rehabilitation, Emory University School of Medicine, Atlanta, GA 30322, USA; ^2^Department of Neurosurgery, Spine Center, Lahey Clinic, Burlington, MA 01805, USA; ^3^Spaulding Rehabilitation Hospital, Harvard Medical School, Boston, MA 02115, USA

## Abstract

*Background*. Among patients with chronic disease, percutaneous endoscopic gastrostomy (PEG) tubes are a common mechanism to deliver enteral feedings to patients unable to feed by mouth. While several cases in the literature describe difficulties with and complications of the initial placement of the PEG, few studies have documented the effects of a delayed diagnosis of a misplaced tube. *Methods*. This case study reviews the hospitalization of an 82 year old male with an inadvertent placement of a PEG tube through the transverse colon. Photos of the placement in the stomach as well as those of the follow up colonoscopy, and a recording of the episodes of diarrhea during the hospitalization were made. *Results*. The records of this patient reveal complaints of gastrointestinal distress and diarrhea immediately after placement of the tube. Placement in the stomach was verified by endoscopy, with discovery of the tube only after a follow up colonoscopy. The tube remained in place after this discovery, and was removed weeks after the diarrhea was unsuccessfully treated with antibiotics. After tube removal, the patient recovered well and was sent home.

## 1. Background

Many patients with chronic disease lack the ability to maintain adequate nutrition by mouth [1]. Percutaneous endoscopic gastrostomy (PEG) tubes are a well-established, effective, and a relatively safe means to deliver enteral feedings to patients unable to feed by mouth [2–4]. In acute rehabilitation hospitals, it is common for a nasogastric (NG) tube to be replaced by a PEG tube to allow for enteral feeding over a long period of time. While surgical gastrostomies have been relatively common, the use of PEG now seems to be the preferred method for those in need of such gastrointestinal access [5–9]. 

There are a number of cases in the literature describing difficulties with and complications of initial placement of PEG tubes, as well as those that develop thereafter [10–19]. For the most part, these complications are realized at the time of initial placement and immediate remedial action is taken. The importance of initial placement has also been shown to be a significant factor in preventing gastric ulcers [5]. Later complications of PEG tube placement include infection at placement site, leaks of the tube or at the insertion site, gastrointestinal (GI) bleeding, and peritonitis [6, 7, 20, 21]. An unusual but very dangerous complication described in the literature is that of the “buried bumper” syndrome [8–10, 22]. This syndrome, which can present early or late, involves the migration of the internal bumper of the PEG tube through or into the abdominal wall resulting in gastrointestinal bleeding, stomach perforation, peritonitis, and, in some cases, even death [11]. In addition to these complications, there have been cases of PEG tube malpositioning through the liver [23–28], duodenum [12], jejunum [29–31], gastric arteries [32], and transverse colon [33–36], with varying ill effects. This case study documents the course of a patient whose PEG tube placement into the stomach was found to involve the inadvertent penetration of the transverse colon with resultant intractable diarrhea. To our knowledge, this represents the first documented case of transverse colonic insertion in the setting of direct visualization by endoscopy.

## 2. Case/Hospital Course

This is a case of an 82-year-old male who suffered multiple traumatic injuries including subdural hematoma after being struck by a motor vehicle while walking on the street. He required intubation and mechanical ventilation for respiratory distress, and was weaned off the ventilator after about one week. The patient was advanced to oral (PO) feedings, but complained of right upper quadrant tenderness. An abdominal ultrasound revealed a normal biliary tree with sludge in the gall bladder. A CT of the abdomen revealed an ileus, and an NG tube was placed. Total parenteral nutrition (TPN) was subsequently initiated for nutritional support. With demonstrated ability to swallow, the patient's NG tube was removed and he was advanced to a dental soft diet. His oral intake was poor at that time, but thought to be improving. With no further surgical or intensive care unit intervention under consideration, the patient was transferred to the medical team at the brain injury unit of the acute rehabilitation hospital.

During his first week at in the brain injury unit, the patient continued to poorly demonstrate PO intake. A nasogastric tube was ordered. The patient was extremely intolerant of the NG, and placement was unsuccessful. A PEG tube was ordered and placed by the gastroenterology team under upper endoscopic visualization. The endoscope was passed under direct visualization. During this procedure the patient had been placed in the supine position and the stomach was insufflated to oppose the gastric and abdominal wall. The abdominal wall was marked and the trocar needle was introduced through the abdominal wall under direct endoscopic view. A snare was introduced through the endoscope and opened in the gastric lumen. The guide wire was passed through the trocar and into the open snare. The snare was closed around the guide wire. A 20 Fr microinvasive gastrostomy tube was tied to the guide wire and pulled through the mouth and into the stomach. The trocar needle was removed and the gastrostomy tube was pulled out from the stomach. The external bumper was attached to the tube and the gastrostomy tube was cut to remove the guidewire. The placement was noted to be uncomplicated. No abnormalities were noted in the gastric wall or the esophagus at the time of placement.

**Figure 1:**
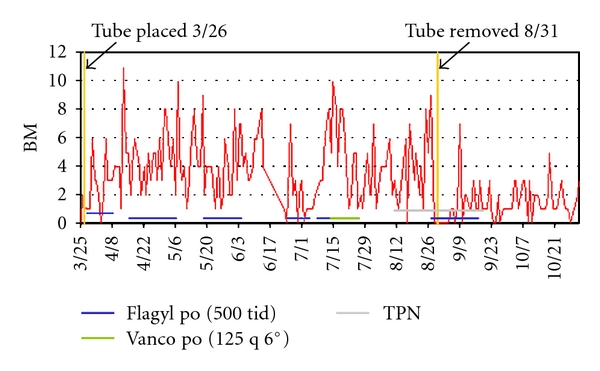
Bowel movement frequency per day for the patient's entire length of stay at a rehabilitation hospital.

During the next week, however, the patient began to develop diarrhea. Dietary modifications did not improve the diarrhea, which proceeded to worsen. The patient's stool tested positive for *Clostridium difficile*. He was started on a course of metronidazole, but showed no improvement. (See Figure 1). The infectious disease team was consulted, and recommendations were made to extend the treatment. Multiple courses of metronidazole were administered over several weeks.

During this time period, the patient underwent both ultrasonographic imaging and laboratory testing to investigate his gastrointestinal distress. These studies revealed a distended gallbladder with sludging and a mildly elevated lipase, respectively, but were otherwise unremarkable. A KUB was performed, which failed to show obstipation. An upper endoscopy was also ordered, and revealed that the G-tube was still in place without evidence of erosion or gastric irritation. An abdominal CT was ordered using IV and oral contrast. These were read as revealing mild perianal inflammatory changes containing air and distal rectal wall thickening with no drainable fluid, and no CT evidence of inflammatory colitis. It was noted that a G-tube was seen in situ. A repeat CT was ordered 6 weeks later, again noting a gastric tube in place with additional comments of no bowel obstruction and perianal collections containing air and fluid. With the development of symptoms including occasional nausea and vomiting, the patient was sent for a head CT, which was unremarkable for new findings.

**Figure 2:**
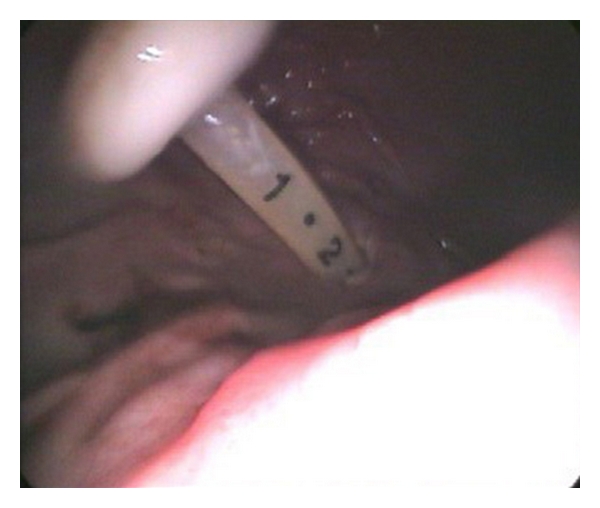
Photograph demonstrating PEG tube termination inside the lumen of the stomach.

**Figure 3:**
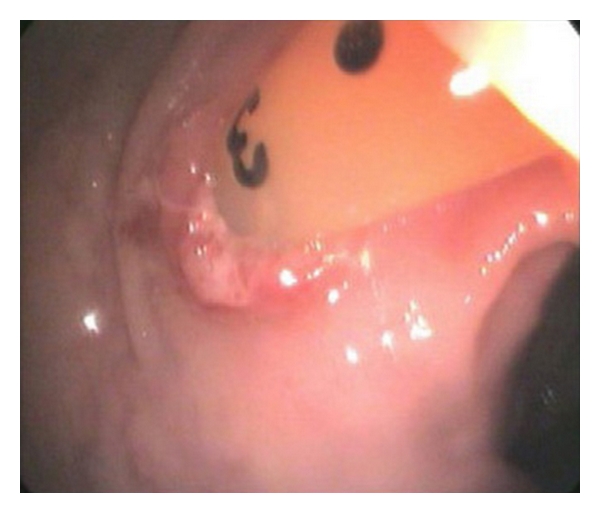
Photograph demonstrating the PEG tube exiting from the wall of the transverse colon.

**Figure 4:**
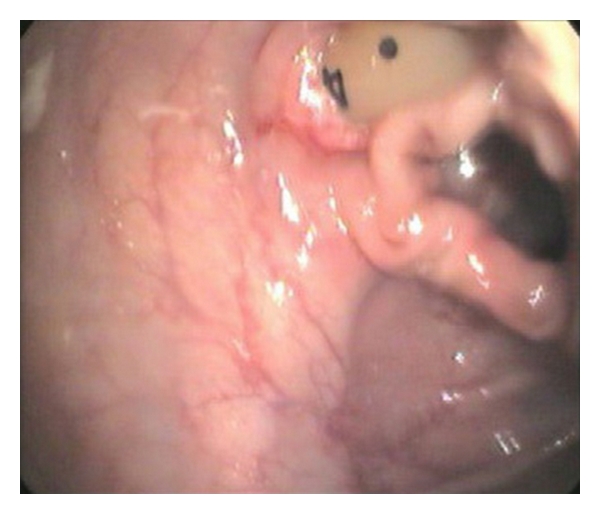
Photograph demonstrating PEG tube entering the wall of the transverse colon.

 The patient had hemoccult-positive stools that were thought to be secondary to hemorrhoids. In spite of this, a colonoscopy was ordered, showing nodular mucosa in the rectum, which was thought to be nonspecific and nondiagnostic for colitis. At that procedure it was discovered that the PEG tube had pierced the transverse colon, entering and exiting it terminating in the stomach (see Figures 2, 3, and 4). No other GI abnormalities were noted. GI surgery was consulted and recommended that the PEG be maintained, as it was thought to be an unlikely source of diarrhea and of no acute concern. The patient continued to have *C. difficile*-positive loose stools. As the diarrhea persisted through four full courses of metronidazole, the infectious disease consultants agreed to switch to PO vancomycin and cholestyramine. These failed to produce improvement in the diarrhea.

Tube feeds were intermittently held while TPN was initiated to rest the bowel. There was interval improvement in the number of stools per day with off-tube feeds, and subsequent resumption of diarrhea once the feeds were restarted. The tube feeds were again held until the patient was reevaluated by GI surgery. With a failure to respond to multiple courses of metronidazole and a course of vancomycin, the patient was sent for a repeat colonoscopy.

 This second colonoscopy revealed the PEG tube had migrated, apparently dislodging from the stomach and coming to rest in the transverse colon. Left unanswered was when this migration had occurred, though a CT two weeks earlier had demonstrated proper placement, with the tube terminating in the stomach. At that colonoscopy, the tube was surgically removed and a new tube was placed without complication. The frequency of the diarrhea immediately declined and the patient's symptoms improved (see Figure 1). A review of the data demonstrated the mean number of diarrheal episodes per day to be 3.8 during the entire period with the “tube in” and 1.5 per day with the “tube out,” for a difference of about 2.4 episodes/day (see Table 1). For a statistical comparison, we calculated a *t*-test assuming—conservatively—that the variances were not equal (*P* < 0.00005). We also did a Wilcoxon rank sum test (see Table 2), as a nonparametric alternative to the *t*-test. Again, the *P* value was found to be statistically significant (*P* < 0.00005). Moreover, the output from this procedure also gives an interesting result shown in the last line of the output: the proportion of pairs where the episode for the “tube in” period is greater than the episodes for the “tube out” period. Pairing each “tube in” day with each “tube out” day yields 8,906 pairs of days. In 80% of these pairs, there are found to be more episodes of diarrhea for the “tube in” days than for the “tube out” days.

 The patient's PEG tube feedings were resumed and the patient did reasonably well. Thereafter, he began to demonstrate improved PO intake and was able to participate in more therapy without diarrhea or excessive weakness. His PEG tube was removed after he maintained adequate oral nutrition. The patient was discharged shortly thereafter to a skilled nursing facility. At the time of followup 6 months later, the patient was living at home with his wife, without any recurrence of previous gastrointestinal complaints.

## 3. Discussion

This case describes a PEG inadvertently placed through the transverse colon and into the stomach, despite intraprocedural direct visualization with an endoscope. Previous cases of malpositioning and colonic migration have been reported, though without the use of intra-procedural endoscopy [7, 8, 15, 30, 33–35, 37–45]. A comparison of the patient's bowel patterns before and after the removal seems to demonstrate that placement of the tube caused an increase in bowel motility and frequency. Although a concurrent bowel infection has been shown to complicate the development of diarrhea or alter its manifestations [46], the data presented in this paper provide reasonable evidence that the PEG tube placement clearly contributed to the diarrhea.

This case seems valuable to the practicing physician for two reasons. First, it demonstrates that even under direct intra-procedural visualization, a PEG tube may pierce other viscera. This suggests that other complications of enteral access [47] may likewise result, despite the employment of putatively excellent preventative measures. This is sobering given the current governmental pressure to defund the treatment of potentially foreseeable complications [48–50]. Second, this study does demonstrate that irritation caused by the piercing of the transverse colon may increase bowel motility and be a cause of recalcitrant diarrhea. To our knowledge, this is the first demonstration of this association. In cases of intractable diarrhea misplacement of the PEG must therefore be added to the physician's differential.

## Figures and Tables

**Table 1:** Two-sample *t*-test with unequal variances.

	Days	Mean stools/day	Std. error	Std. deviation	(95% conf. interval)	
With G-tube	146	3.835616	.2031931	2.455191	1.434014	4.237219
Without G-tube	61	1.47541	.1662945	1.298801	1.142771	1.808048
Combined	207	3.140097	.1687944	2.428529	2.807311	3.472883

Satterthwaite's degrees of freedom = 193.982.

*P* < 0.0005.

**Table 2:** Two-sample Wilcoxon rank-sum (Mann-Whitney) test.

	Days	Rank sum	Expected
With G-tube	146	17861	15184
Without G-tube	61	3667	6344
Combined	207	21528	21528

*P* < 0.00005.
